# Host Responses in Life-History Traits and Tolerance to Virus Infection in *Arabidopsis thaliana*

**DOI:** 10.1371/journal.ppat.1000124

**Published:** 2008-08-15

**Authors:** Israel Pagán, Carlos Alonso-Blanco, Fernando García-Arenal

**Affiliations:** 1 Departamento de Biotecnología, E.T.S.I. Agrónomos and Centro de Biotecnología y Genómica de Plantas, Universidad Politécnica de Madrid, Madrid, Spain; 2 Departamento de Genética Molecular de Plantas, Centro Nacional de Biotecnología, Consejo Superior de Investigaciones Científicas (CNB-CSIC), Madrid, Spain; The Pennsylvania State University, United States of America

## Abstract

Knowing how hosts respond to parasite infection is paramount in understanding the effects of parasites on host populations and hence host–parasite co-evolution. Modification of life-history traits in response to parasitism has received less attention than other defence strategies. Life-history theory predicts that parasitised hosts will increase reproductive effort and accelerate reproduction. However, empirical analyses of these predictions are few and mostly limited to animal-parasite systems. We have analysed life-history trait responses in 18 accessions of *Arabidopsis thaliana* infected at two different developmental stages with three strains of *Cucumber mosaic virus* (CMV). Accessions were divided into two groups according to allometric relationships; these groups differed also in their tolerance to CMV infection. Life-history trait modification upon virus infection depended on the host genotype and the stage at infection. While all accessions delayed flowering, only the more tolerant allometric group modified resource allocation to increase the production of reproductive structures and progeny, and reduced the length of reproductive period. Our results are in agreement with modifications of life-history traits reported for parasitised animals and with predictions from life-history theory. Thus, we provide empirical support for the general validity of theoretical predictions. In addition, this experimental approach allowed us to quantitatively estimate the genetic determinism of life-history trait plasticity and to evaluate the role of life-history trait modification in defence against parasites, two largely unexplored issues.

## Author Summary

Hosts have developed a variety of mechanisms to compensate for the negative impact of parasite infection. Modification of life-history traits in response to parasitism has received less attention than other defence strategies. Life-history theory assumes trade-offs between resource allocation to different fitness components, and predicts that hosts under parasitism will allocate more resources to reproduction, subtracting them from those dedicated to growth and survival. Empirical support for predictions is not abundant, and derives mostly from the analysis of animal-parasite systems. We have analysed the modification of various life-history traits in the plant *Arabidopsis thaliana* infected by *Cucumber mosaic virus*. Life-history trait modification upon virus infection depended on the host genotype and on the developmental stage at infection. All plant genotypes delayed flowering, but only the more tolerant ones allocated more resources to reproduction, and reduced the length of reproductive period. These results agree with reports from parasitised animals and with predictions from life-history theory, providing empirical support for the general validity of theoretical predictions. In addition, results allow for the more precise evaluation of the role of life-history trait modification in defence against parasites by taking into account plant–virus interactions where life-history traits were differentially modified.

## Introduction

Parasites affect the welfare of humans and of domestic animals and plants, with a high socioeconomic impact. In addition, an increasing number of reports provide evidence of the important role of parasites in ecosystem composition and dynamics [1]. Parasite infection has a negative impact on host fitness, which has been defined as virulence [2]. Consequently, parasites may modulate the dynamics and genetic structure of populations of their hosts, as well as of non-host species by altering inter-specific competition [3],[4]. It has been proposed that, through these effects, parasites may drive biodiversity [5]. Knowing how hosts respond to parasite infection is capital for understanding the role of parasites in shaping host populations and ecosystems. Hosts have developed a variety of mechanisms to compensate for the cost of parasite infection, which may be grouped into four strategies [6]: hosts can modify their behaviour to avoid contact with parasites; hosts may have mechanisms that prevent the establishment of infection and trigger defence responses; hosts may develop immune systems, which in addition to act as barriers to infection may also clear the infection if parasites overcome host defences; and a fourth mechanism to reduce the harm of parasite infection is tolerance, which may involve the alteration of host life-history traits. While literature on the first three strategies is extensive, particularly regarding defence responses and immune systems, tolerance and, particularly, host life-history trait modification, has received comparatively less attention.

Various host life-history traits have been reported to respond to pathogen infection, including pre-reproductive life span [7],[8], reproductive effort [9],[10], and body size [11],[12]. These observations have prompted theoretical analyses aimed to predict optimal host life-history trait responses to parasitism. Life-history theory makes predictions for the evolution of resource investment by organisms, based on the concept that trade-offs exist between resources allocated to different fitness components: reproduction, growth and survival [13]. The optimal pattern of resource allocation may differ depending on environmental conditions, which include parasitism [14]. Thus, parasite infection may modify optimal resource distribution. Inspired by this concept, models for evolution of resource allocation predict that parasitised hosts will allocate more resources to reproduction, subtracting them from those dedicated to growth and survival [15]–[18]. Life-history theory also states that environmental conditions affecting mortality rates modify temporal life-history schedules in order to maximize fitness [19]. Accordingly, models predict that highly virulent parasites will induce shorter host pre-reproductive periods in order to produce progeny before resource depletion, castration or death. In contrast, low virulence will result in a delay in host reproduction, which allows for compensation of parasite damage [20],[21].

If theoretical efforts at understanding the evolution of life-history traits under parasite infection are not abundant, experimental analyses are scarcer and have been mostly limited to animal hosts and highly virulent parasites causing mainly host death or castration. Most experimental results support predictions for the effects of parasitism on age at maturity [7],[22] or on reproductive effort [23],[24]. However, there are also examples that do not fit theoretical predictions and that have been explained as consequence of particular host genetic features [8],[25], environmental conditions [26] or parasite manipulation of the trade-off between growth and fecundity (e.g. gigantism) [11]. Experimental analyses are usually focused on a single host genotype infected by one or various pathogen genotypes, but the role of genotype×genotype interactions, which may affect the outcome of host-parasite interactions [27],[28], has been mostly overlooked. Experimental analyses of the evolution of plant life-history traits under parasitism are rather limited, with the notable exception of analyses of the effects of infection by the fungus *Microbotryum violaceum* on the perennial plant *Silene latifolia* [29],[30]. However, studies of plant host-parasite systems are relevant to test the general validity of theoretical predictions, since plants and animals differ widely in organisation, and plant parasites mostly affect growth and reproduction of their host without causing immediate host death.

To analyse the effects of parasitism on plant life-history traits we have chosen the plant-virus system *Arabidopsis thaliana* L. Heynh. (*Brassicaceae*)-*Cucumber mosaic virus* (CMV, *Bromoviridae*). In the last twenty years, *Arabidopsis thaliana* (from here on, referred to as *Arabidopsis*) has arisen as the model organism for the molecular and genetic study of a wide range of plant traits, including resistance patterns against parasite infection [31],[32]. Recently it has been also used in analyses of host-parasite co-evolution [33],[34] and of life-history traits responses to abiotic stress associated with changes in light, nutrients or water availability [35]–[37]. The annual plant *Arabidopsis* is a typical semelparous species, with two clearly differentiated developmental phases or periods in its post-embryonic life cycle. During the vegetative growth period, a rosette of leaves is produced. Vegetative growth ceases when the vegetative meristem becomes an inflorescence meristem [38]. This is the start of the reproduction period, when the reproductive structure (inflorescence) grows, new flowers are produced continuously and older flowers develop into fruits (siliques). Flower production almost ceases after ripening of the first silique, and most flowers produced later on fail to set fruits and seeds [39],[40]. Plant senescence and death end the reproduction period. Hence, vegetative growth effort, total reproductive effort and progeny production are easily differentiated in *Arabidopsis.*

CMV is a generalist virus that infects about 1,200 plant species in more than 100 mono- and dicotyledonous families, including natural populations of *Arabidopsis* (our unpublished observations). CMV is horizontally transmitted by more than 70 species of aphids, and vertically through seeds with rates that vary depending on the genotypes of CMV and host-plant species. CMV has a messenger-sense, single-stranded, three-segmented RNA genome encapsidated in three isometric particles. The structure of the CMV genome and the roles of the five encoded proteins have been extensively characterized. The genetic variability of CMV has also been much analysed and CMV isolates have been classified into two subgroups, subgroup I and subgroup II, based on the nucleotide sequence similarity of their genomes (reviewed in [41],[42]).

In this work, we have tested predictions of life-history evolution theory by analysing the effect of CMV infection on *Arabidopsis* growth and reproductive effort and on age at maturity and reproductive period span. To test the contribution of genotype×genotype interactions on life-history traits response to virus infection, we challenged 18 wild genotypes (accessions) of *Arabidopsis* with three CMV strains. A general reduction of growth and reproductive effort was observed after infection as well as a tendency to increase the age at maturity. However, some accessions previously shown to manifest tolerance to CMV infection [34] presented a relative increase of the reproductive effort upon viral infection together with a reduction of the reproductive period. Overall, these life-history trait modifications can be interpreted as host reactions that reduce the impact of infection on plant fitness.

## Results

### Resource allocation to growth and reproduction in *Arabidopsis* accessions

Plant architecture and, consequently, resource allocation to growth and reproductive effort, differ among *Arabidopsis* accessions and condition responses to viral infection [34]. To properly evaluate the effect of virus infection on different fitness components of the host, we first analysed the relationship between rosette weight (*RW*), as a measure of growth effort; inflorescence weight (*IW*) as measure of total reproductive effort; seed weight (*SW*), as a measure of progeny production [43], and total above-ground biomass (*BM*) in mock-inoculated plants of eighteen *Arabidopsis* accessions (see Materials and Methods and Table S1). *SW* was taken as measure of progeny production since it was previously shown that in these accessions CMV infection did not affect seed size or viability [34]. All traits differed significantly among accessions (*P*<1×10^−5^). Rosette weight was positively correlated with inflorescence weight (*r* = 0.61, *P* = 1×10^−4^) and negatively with seed weight (*r* = −0.36, *P* = 0.04), which indicates a general positive correlation between growth and reproductive efforts. No significant correlation was found between inflorescence weight and seed weight (*r* = 0.22, *P* = 0.21).

**Figure 1:**
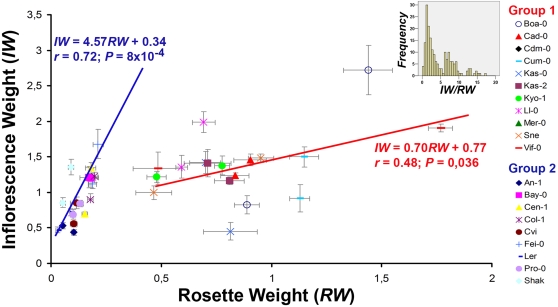
Relationship between growth (*RW*) and reproductive effort (*IW*) in *Arabidopsis* accessions. Correlation between *IW* and *RW* for allometry group 1 (red) and allometry group 2 (blue) using mean accession values of mock-inoculated plants. Data are mean±standard error of *RW* and *IW* in g. The upper-right panel shows the frequency distribution of the *IW/RW* relationship of the 18 accessions, based in individual plant values.

The balance between growth and reproductive effort estimated as *IW/RW,* showed a bimodal distribution across accessions (Figure 1). Thus, two allometric groups of accessions differing significantly in *IW/RW* (*P*<1×10^−5^) were defined: group 1 with *IW/RW* <5.0 (mean value of 1.75±0.17) including accessions Boa-0, Cad-0, Cum-0, Kas-0, Kas-2, Kyo-1, Ll-0, Sne and Vif-0, and group 2 with *IW/RW* >5.0 (mean value 6.99±0.88) containing An-1, Bay-0, Cen-1, Col-1, Cvi, Fei-0, L*er*, Pro-0 and Shak. A differential linear relationship between *RW* and *IW* was found for each group (Figure 1). These two accession groups are the same as those defined in Pagán et al. [34] based on the *SW/BM* relationship.

For each allometric group, *BM* was positively correlated with *RW*, *IW* and *SW* (*r*>0.53, *P*<0.03)*; RW* was positively correlated with *IW* (*r*>0.47, *P*<0.04) but did not correlate with *SW*, and *IW* was positively correlated with *SW* (*r*>0.62, *P*<0.01). Thus, in both allometric groups there is a positive correlation between growth and reproductive efforts and between reproductive effort and progeny production.

### Effects of CMV infection on resource allocation to growth and reproduction

Eighteen *Arabidopsis* were inoculated with three CMV isolates early in the vegetative period (see Materials and Methods). The effect of CMV infection on *Arabidopsis* growth and reproductive efforts was quantified as the ratios of rosette and inflorescence weights, respectively, between infected and mock-inoculated plants (*RW_i_*/*RW_m_* and *IW_i_*/*IW_m_*, *i* and *m* denoting infected and mock-inoculated plants respectively) (see Table S2 for statistical parameters of the variables). A general reduction of *RW* and *IW* was observed in infected plants, but the effect of CMV infection on both traits depended on the accession, isolate, and the interaction between the two genotypes (*P*<1×10^−5^) (see Table S3 for ANOVA parameter values). On average, the effect of infection by Fny-CMV on both *RW* and *IW* was about 16% stronger than the effect of infection by LS-CMV and about 38% stronger than the effect of De72-CMV (Figure S1, and Table S2). Broad-sense heritabilities of *RW_i_*/*RW_m_* ranged from low to moderate (*h^2^_b_* = 0.11–0.56) depending on isolate, while *IW_i_*/*IW_m_* showed a narrower variation (*h^2^_b_* = 0.39–0.52) (Table S2). Isolates and accessions accounted for a higher fraction of variance of *RW_i_*/*RW_m_* than the interaction (*VC* = 16.92, *VC* = 17.95, *VC* = 6.15, respectively) but the three components explained similar levels of *IW_i_*/*IW_m_* variance (*VC* = 22.22, *VC* = 19.14, *VC* = 16.67 for isolate, accession and interaction, respectively) (Table S3). Thus, responses of *Arabidopsis* on growth and reproductive efforts to CMV infection depend on the host-genotype×parasite-genotype combination.

**Figure 2:**
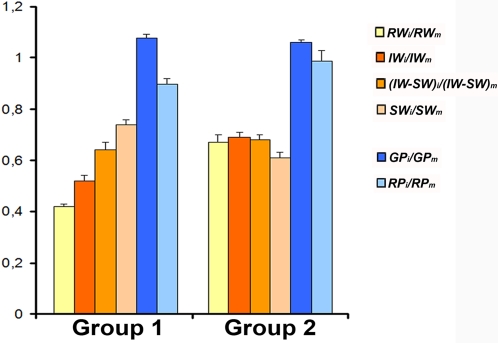
Effect of CMV infection on life-history traits for the two allometric groups of accessions. Effect of viral infection was estimated as the ratio between infected (*i*) and mock-inoculated (*m*) plants. Data are mean±standard errors of accession means.

When the two allometric groups of accessions were compared, they differed significantly for *RW_i_*/*RW_m_* and *IW_i_*/*IW_m_* (*P*<0.009) indicating that the effect of virus infection depends on the allometric relationships (Table S4). Isolate and group explained a similar and higher level of *RW_i_*/*RW_m_* and *IW_i_*/*IW_m_* variation than the interaction accession×isolate (e.g. *VC* = 16.92, *VC* = 11.11, *VC* = 0.97, respectively, for *RW_i_*/*RW_m_*). Therefore, both allometric groups were analysed separately. As shown in Figure 2, the effect of infection was much larger for accessions of group 1 (0.42±0.01 and 0.52±0.02 for *RW* and *IW*, respectively) than for group 2 (0.67±0.03 and 0.69±0.02 for *RW* and *IW*, respectively). For accessions of group 1 the effect of infection on *RW* was 19% larger than on *IW* (*P*<2×10^−5^), but the effects were similar for accessions of group 2 (*P*≥0.61) (Figure 2). Thus, the effect of virus infection on growth and reproductive efforts depends on the allometric relationship of the accessions.

**Figure 3:**
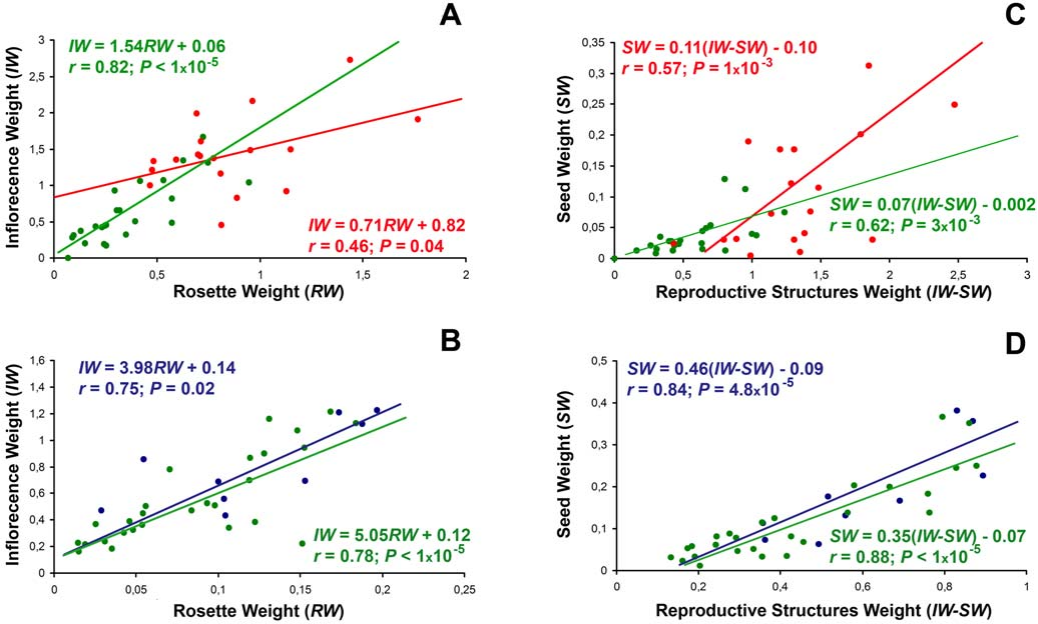
Effects of CMV infection on growth/reproduction resource allocation of *Arabidopsis* accessions. (A) Effect of infection on *IW/RW* relationship for allometry group 1. (B) Effect of infection on *IW/RW* relationship for allometry group 2. (C) Effect of infection on *SW/(IW-SW)* relationship for allometry group 1. (D) Effect of infection in *SW/(IW-SW)* relationship for allometry group 2. Relationship in infected plants (green) is compared with that of mock-inoculated plants of allometry group 1 (red) and 2 (blue). Data are mean values of each accession. *RW*, *IW* and *(IW-SW)* units are g.

The effect of infection on the relationship between growth and reproductive efforts was further analysed using the ratio *(IW/RW)_i_/(IW/IRW)_m_*. Significant differences were found among allometric groups, isolates and due to the interaction between both factors (*P*<0.007) (Table S4). Therefore, the effect of CMV infection on *IW*/*RW* was analysed for each accession group separately. For allometric group 1, linear regressions of *IW* on *RW* significantly differed in slope and intercept between infected and mock-inoculated plants (*P*<0.01) and the average value of *IW/RW* was higher for infected than for mock-inoculated plants (2.75±0.02 *vs*. 2.11±0.16) (Figure 3A). For group 2 of accessions, the regression lines of *IW* on *RW* did not differ significantly between mock-inoculated and infected plants (*P*≥0.37), for which average values of *IW/RW* were 8.03±0.69 and 7.52±0.47, respectively (Figure 3B). Therefore, infected plants of allometry group 1, but not of group 2, allocated a higher fraction of resources to reproduction than mock-inoculated plants.

### Effects of CMV infection on resource allocation to reproductive structures and progeny production

CMV effects on the weights of seeds and reproductive structures were quantified as the ratios of infected *vs.* mock-inoculated plants *SW_i_/SW_m_* and *(IW-SW)_i_/(IW-SW)_m_*, respectively (see Table S2 for statistical parameters). Viral effects on *SW* and *IW-SW* differed significantly between isolates and accessions and for the isolate×accession interaction (*P*<1×10^−5^; Table S3). Broad-sense heritabilities of *SW_i_/SW_m_* were lower (*h^2^_b_* = 0.19–0.31) than those of *(IW-SW)_i_/(IW-SW)_m_* (*h^2^_b_* = 0.34–0.44) (Table S2). Accession factor explained a higher fraction of the variation of *SW_i_*/*SW_m_* and *(IW-SW)_i_/(IW-SW)_m_* than isolate or the interaction accession×isolate (e.g. *VC* = 2.28, *VC* = 28.92, *VC* = 3.01, for isolate, accession and interaction for *SW_i_*/*SW_m_*) (Table S3). In addition, virus infection had the same effect on *SW* and *IW-SW* (*P* = 0.52), average values of *SW_i_/SW_m_* and *(IW-SW)_i_/(IW-SW)_m_* being 0.67±0.01 and 0.66±0.02. Thus, CMV effect on progeny production and on reproductive structures depended on host-genotype×parasite-genotype interaction.

The effect of CMV on *SW*, but not on *(IW-SW)*, differed significantly between the two allometric groups (*P*<3×10^−4^ and *P*>0.23, respectively). Isolate, group and the interaction accounted for a similar fraction of *SW_i_/SW_m_* variation (e.g. *CV* = 2.28; *CV* = 5.51; *CV* = 1.91 for isolate, group and isolate×group interaction, respectively), whereas isolate and the interaction isolate×group accounted for a similar fraction of *(IW-SW)_i_/(IW-SW)_m_* variance (*CV* = 3.39; *CV* = 2.12 for isolate and isolate×group interaction, respectively) (Table S4). Therefore, the two allometric groups were analysed separately. As shown in Figure 2, the effect of virus infection on *SW* was smaller for group 1 (0.74±0.02) than for group 2 (0.61±0.02), but no significant difference was found for *(IW-SW)_i_/(IW-SW)_m_* (0.68±0.02 and 0.64±0.03, for group 1 and 2, respectively). In addition, for accessions of group 1, *SW* was significantly less affected by CMV infection than *IW-SW* (8%, *P*<3.7×10^−2^). The opposite was observed for group 2, where viral effects on *SW* were slightly higher than on *IW-SW* (3%, *P*<4.3×10^−2^). Thus, viral effect on seed and reproductive structures weight also depended on plant architecture.

The relationship between seed weight (*SW*) and reproductive structure weight (*IW-SW*) was further analysed using the ratio [*SW*/(*IW-SW*)]*_i_*/[*SW*/(*IW-SW*)]*_m_*. This ratio showed strong significant differences among accessions (*P*<1×10^−5^; Table S3) and between allometric groups (*P* = 6×10^−4^; Table S4) but no or small differences among isolates (Tables S3 and S4). Hence the effect of infection on *SW*/(*IW-SW*) was analysed for each accession group separately. For group 1 of accessions, *SW* to *IW-SW* regression lines of infected plants (Figure 3C) differed significantly from those of mock-inoculated plants (*P* = 0.03), the average value of *SW*/(*IW-SW*) being 0.07±0.001 for mock-inoculated plants, and 0.09±0.001 for infected plants (Figure 3C). For group 2, regression lines did not differ between infected and mock-inoculated plants (*P*>0.28), and *SW*/(*IW-SW*) showed average values of 0.28±0.02 and 0.25±0.02 for mock-inoculated and infected plants respectively (Figure 3D). Thus, infected plants of allometry group 1, but not of group 2, allocated proportionally more resources to the production of progeny than to reproductive structures than did healthy plants.

### Effect of CMV infection on age at maturity and reproductive life span

To analyse if the temporal control of *Arabidopsis* transition from vegetative growth to reproductive phase may vary in response to CMV infection, we measured the span of growth and reproductive periods (*GP* and *RP*, respectively). Both traits differed significantly among accessions in mock-inoculated plants (*P*<1×10^−5^). In addition, *GP* was negatively correlated with *RP* when all accessions were analysed together and for each allometric group of accessions (*r*>−0.32, *P*<0.04). *Arabidopsis* heritabilities of temporal life-history traits and their CMV responses ranged from low to moderate (*h^2^_b_* = 0.14–0.36) depending on CMV isolate (Table S2).

**Figure 4:**
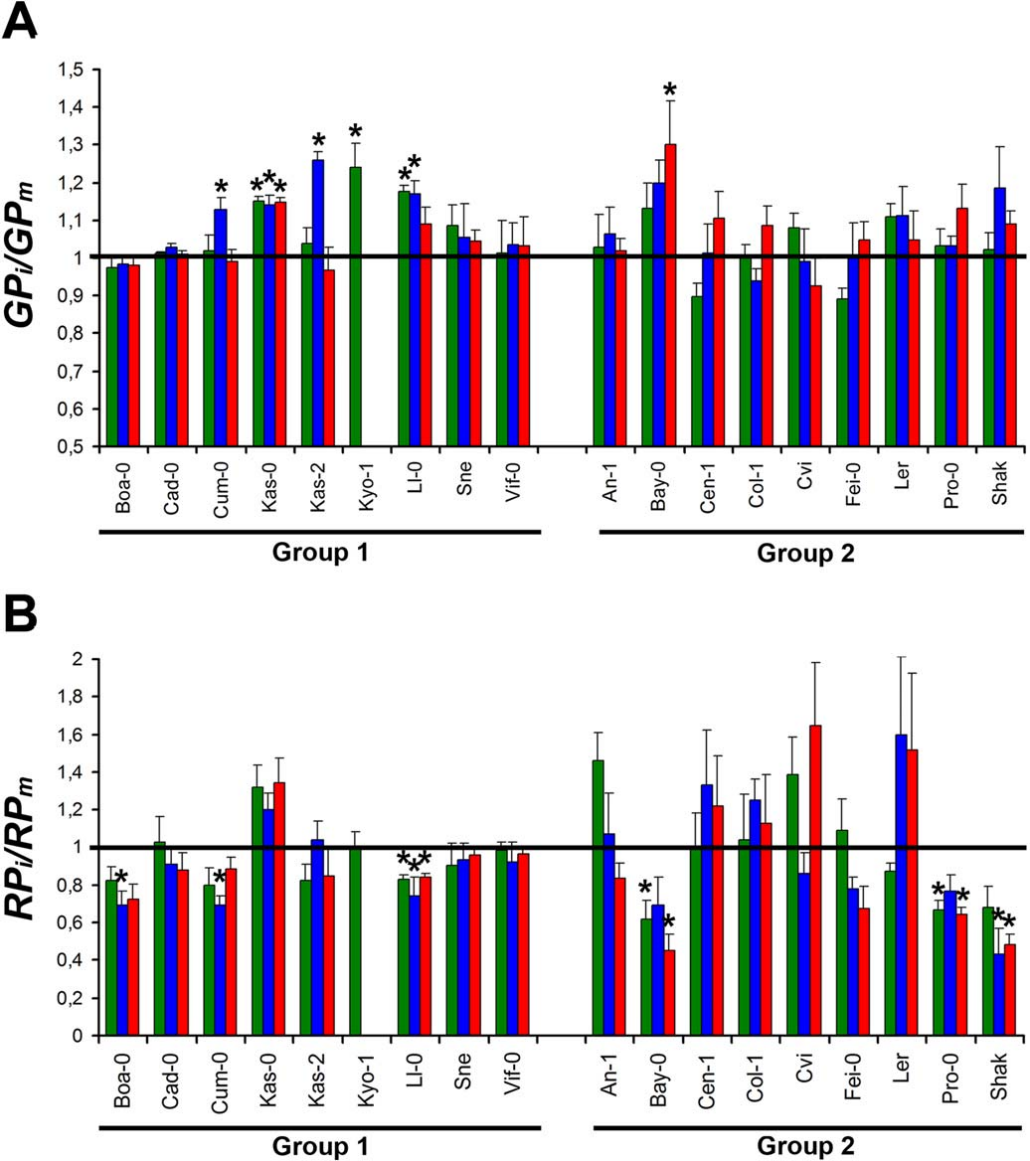
Effect of viral infection on growth (*GP*) and reproductive (*RP*) period span of *Arabidopsis* accessions. (A) Effect of CMV infection in *GP* period span estimated as *GP_i_*/*GP_m_*, where *i* and *m* denote infected and mock-inoculated plants, respectively. (B) Effect of CMV infection in *RP* period span estimated as described for (A). Data are mean values of accessions infected by each CMV isolates±standard errors. Asterisks indicate significant differences between mock-inoculated and infected plants (*P*<0.05). The effect of infection is shown for LS-CMV (green), Fny-CMV (blue) and De72-CMV (red). Accessions are divided into allometry groups 1 and 2.

The effect of virus infection on *GP* and *RP* was quantified as *GP_i_/GP_m_* and *RP_i_/RP_m_*. Both traits showed significant differences among accessions (*P*<1×10^−5^) and due to the interaction accession×isolate (*P* = 0.01), but not among isolates (*P* = 0.74). Again, accessions accounted for a higher proportion of the variance than the interaction (e.g. *VC* = 17.27; *VC* = 9.39, for accessions and interaction, respectively) (Table S3). However, CMV infection affected differently *GP* and *RP*. Infection resulted in an increase of *GP* in most accessions (16 out of 18), although significant differences were observed in six of them (Cum-0, Kas-0, Kas-2, Kyo-1, Ll-0 and Bay-0) (Figure 4A). In contrast, infection decreased *RP* in 12 out of 18 accessions, the decrease being significant in six of them (Boa-0, Cum-0, Ll-0, Bay-0, Pro-0 and Shak) (Figure 4B). We further analysed the effect of infection on the time span to seed production (*GP*+*RP*), and again significant differences were found among accessions and for the interaction isolate×accession (*P*<5×10^−3^; *VC* = 12.12, *VC* = 6.06 for accession and interaction, respectively) but not among isolates (*P*>0.31) (Table S3). Infection resulted in shortening of *GP*+*RP* in 11 accessions and elongation in 7 out of 18 accessions, although differences with mock-inoculated controls were significant only for 4 out of 11 (Boa-0, Cum-0, Fei-0 and Shak) and 3 out of 7 accessions (Kas-0, Kyo-1 and L*er*) respectively (not shown). All accessions showing reduction of *RP* also had shorter *GP*+*RP*, except Kyo-1.

Comparison of CMV effects on temporal life span traits between the two allometric groups of accessions showed significant differences for *RP_i_/RP_m_ (P* = 1×10^−3^). Groups 1 and 2 showed mean values 0.90±0.02 and 0.99±0.04 respectively (Figure 2), allometric group accounting for a 5.1% of *RP_i_/RP_m_* variance (Table S4). No significant difference between groups was found for *GP_i_/GP_m_* (*P* = 0.29), group 1 and 2 showing mean values of 1.08±0.01 and 1.06±0.01, respectively. On the other hand, (*GP*+*RP)_i_/(GP+RP)_m_* differed among groups (*P* = 2×10^−3^), which showed mean values of 0.98±0.01 and 1.02±0.01 for group 1 and group 2, respectively. Overall, upon CMV infection, most *Arabidopsis* genotypes tended to increase the age at maturity (growth period). In contrast, the effect on reproductive life span and time to seed production depended on the accession allometric group, both traits being increased in accessions of group 2.

### Relationship between the effects of CMV infection on temporal life-history traits and on resource allocation

To explore if the amount of resources allocated to growth and reproduction might condition the span of growth and reproductive periods, we analysed the relationship between both sorts of traits. In mock-inoculated plants, when all accessions were analysed together, duration of growth period was positively correlated with plant biomass and rosette weight (*r* = 0.37, *P* = 0.03; *r* = 0.57, *P* = 1×10^−3^); it was negatively correlated with seed weight (*r* = −0.54, *P* = 1×10^−3^); and it did not correlate with inflorescence weight (*r* = 0.08, *P* = 0.64). Reproductive period was positively correlated with biomass and inflorescence weight (*r* = 0.38, *P* = 0.03; *r* = 0.43, *P* = 0.01 respectively), but not with rosette and seed weights (*r* = 0.27, *P* = 0.14; *r* = 0.19, *P* = 0.28, respectively). In contrast, no significant correlation was found between the effect of CMV infection on the amount of resources allocated to vegetative or reproductive structures (*RW_i_/RW_m_* and *IW_i_/IW_m_*,) and on the time invested in vegetative and reproductive growth (*GP_i_/GP_m_* and *RP_i_/RP_m_*, respectively) when all accessions and isolates were analysed together (*r*≤−0.13, *P*≥0.32). Similarly, no significant correlation was found when the various traits were analysed separately for each viral isolate (*r*≤0.47, *P*≥0.08).

When these relationships were analysed for each accession separately, four of them (Cad-0, Cum-0 from group 1, and Bay-0 and Shak, from group 2) showed significant negative correlation between *RP_i_/RP_m_* and *IW_i_/IW_m_* (*r*≥−0.43; *P*≤0.03); five accessions (An-1, Col-1, Cvi, Fei-0 and Shak all from group 2) presented a significant positive correlation between *GP_i_/GP_m_* and *RW_i_/RW_m_* (*r*≥0.67; *P*≤0.01); and in three accessions (Cum-0, Kas-2 and Ll-0 from group 1) *GP_i_/GP_m_* was negatively correlated with *IW_i_/IW_m_* (*r*≥−0.45; *P*≤0.02).

The relationships between *GP_i_/GP_m_* or *RP_i_/RP_m_* and *SW_i_/SW_m_* or *(IW-SW)_i_/(IW-SW)_m_*, were also analysed. When all accessions were considered together, viral effects in *RP* and *SW* were marginally correlated (*r* = −0.24; *P* = 0.07). No other significant correlation was found when considering all accessions (*r*≤−0.11; *P*>0.39). When each allometric group of accessions was analysed separately, a marginal negative correlation was found between *RP_i_/RP_m_* and *SW_i_/SW_m_* for group 1 (*r* = −0.33; *P* = 0.06) but not for group 2 (*r* = −0.21; *P* = 0.37). CMV effect on *GP* was positively correlated with viral effects on *SW* and *IW-SW* in group 2 (*r* = 0.35; *P* = 0.04), but not in group 1 (*r* = 0.15; *P* = 0.53). *RP_i_/RP_m_* and *(IW-SW)_i_/(IW-SW)_m_* were not correlated for any allometric group (*r*≤0.18; *P*≥0.16).

Thus, virus infection disrupted the relationships between resource allocation and temporal life-history traits that occurred in mock-inoculated plants.

## Discussion

Plastic modification of life-history traits in response to environmental conditions may be an adaptive mechanism to selection pressures such as abiotic stress, intra- or inter-specific competition or parasitism/disease [15],[44],[45]. Parasites are important ecological agents that can mediate changes in host life-history traits by two sorts of mechanisms. On one hand, parasitic use of host resources can lead to modifications of host resource allocation and developmental time schedules as pathogenic effects of parasitism. Alternatively, life-history modifications may be host responses to compensate for the negative effects of parasitism [17],[18],[20],[21]. The latter are then considered part of tolerance mechanisms, since tolerance is defined as the host ability to reduce the effect of infection on its fitness [46].

Previously, we have reported that within host multiplication of CMV in *Arabidopsis* does not correlate with virulence due to accession-specific tolerance mechanisms associated with differences in resource allocation patterns [34]. These results prompted to study if *Arabidopsis* shows plastic responses of life-history traits to CMV parasitism, as a CMV tolerance mechanism. In this work, we have analysed the effects of CMV parasitism on several *Arabidopsis* life-history traits related with resource allocation and life cycle schedule and found significant plastic modifications of most of them. The relationships among life-history traits has been widely analysed in plants, and correlations between age at maturity and growth effort, and between reproductive period span and reproductive efforts are well documented [47]–[49]. These significant correlations also occurred in our mock-inoculated plants, but not in the infected ones, which indicates that CMV infection not only modifies life-history traits but also alter the relationships between them.

Virus infection had a major effect on resource allocation to growth and reproduction, infection resulting in a general reduction of resources allocated to both traits. However, allocation of resources upon infection was different depending on the allometric features of *Arabidopsis* genotypes. In accessions of group 1, with a low ratio inflorescence weight (*IW*) to rosette weight (*RW*), infection at an early vegetative stage modified the pattern of resource allocation at two levels. First, vegetative growth of infected plants was severely reduced, but a larger fraction of resources was allocated to reproduction than to growth when compared with mock-inoculated plants (Figure 2). Second, infected plants allocated a higher fraction of resources than mock-inoculated ones to progeny production than to production of reproductive structures (Figure 2). In a second experiment, in which *Arabidopsis* accessions were inoculated at the beginning of the reproductive stage (see Methods), similar results were obtained, although the effect of infection on growth and reproductive efforts was less severe and the *IW/RW* relationship was not significantly altered. However, the fraction of resources allocated to progeny production was also increased relative to that allocated to reproductive structures (data not shown). In contrast, the effect of infection on accessions of allometric group 2, with a high ratio of inflorescence weight to rosette weight, did not result in significant modifications of resource allocation neither when plants were infected at vegetative stage, nor at the beginning of the reproductive stage (not shown). The shorter life cycles and the higher fraction of reproductive vs. total biomass characteristic of accessions of allometric group 2 [34] could reduce their ability to modify resource allocation upon infection.

Temporal life cycle parameters of *Arabidopsis* also responded to CMV infection but effects were much smaller than those observed for resource allocation. Vegetative and reproductive span traits behaved differently. Plants infected at vegetative stage tended to increase growth period span (*GP*) by delaying flowering time, independently of allometric group (Figure 2). On the other hand, changes in reproductive span (*RP*) differed significantly between the two allometric groups. Early CMV infection of accessions of group 1 resulted in a reduction of *RP* and total time to seed production (*GP+RP*) indicating faster reproduction of infected plants. These effects were not observed in plants of accession group 2, which were less tolerant to CMV infection [34].

Modification of life-history traits in parasitised hosts can be part of a host defence response, or may be due to the pathogenic effects of parasitism, either as a manipulation of the host by the parasite, which derives some advantage from it, or a by product of infection [7],[16],[20]. However, causal distinction of life-history modifications is not straightforward. Elongation of *GP* and/or inability for reproduction has been interpreted in parasitised insects and molluscs as due to parasite manipulation [50]–[52], as a retard/arrest in development would favour parasite transmission (but see also [53]). However, it seems unlikely that the observed increase of *GP* will be the result of a CMV modification of *Arabidopsis* life cycle favouring its transmission because aphids that transmit CMV [42] can acquire the virus from any green organ, and the total *(GP+RP)* was often shortened by infection. It has been shown that *Arabidopsis* can modulate rosette growth in response to resource availability to maximize reproduction later in development [37]. Since CMV infection results in diminished growth, it can be speculated that plants will delay flowering until a minimum rosette size is attained. Hence, the increase of GP might be interpreted as a by-product of parasitism, although it cannot be discarded that it is part of a general tolerance defence reaction.

Our experimental approach do not allow to determine if resource allocation responses of *Arabidopsis* are a defensive mechanism triggered by the host in order to reduce the impact of CMV infection in its fitness, or an unavoidable consequence of the virus pathogenic effects. These two possibilities could be analysed by mimicking viral infection but avoiding parasite multiplication. It has been reported for several plant species, including *Arabidopsis*, that expression of different virulence factors in transgenic plants induces viral-like symptoms in the absence of infection (e.g., [54]–[58]). However, life-history trait modifications were not analysed in these transgenic plants, which would determine if the host plant activates compensatory mechanisms in response to virus damage or if resource allocation modifications are due to the viral multiplication. Despite this uncertainty, our results support the hypothesis that life-history trait modifications are a defence mechanism in response to CMV infection. The modification of resource allocation in accession group 1 but not in accession group 2 correlates with the lower virulence of CMV on accession group 1 [34] and might partly explain the tolerance to CMV infection observed in this group of accessions as compared with those of group 2. Thus, when infected late in the life cycle, plants of group 1 suffer less from infection than plants from group 2 (data not shown), but when infected early during vegetative development, the growth of plants from group 1 is more severely reduced than the growth of plants from group 2, although tolerance results in a less severe effect of infection on progeny production. The accession group explained ∼5%–10% of the variance of the effect of infection on *RW*, *IW*, *RW/IW* ratio and SW, and a similar fraction of the variance of virulence (effect of infection on progeny production). These results strongly suggest that the differential CMV tolerance observed between both *Arabidopsis* allometric groups is due to the distinct plastic responses of resource allocation traits. Faster reproduction of infected plants of group 1, but not of group 2, appears also associated with tolerance to CMV. Ultimately, identification and characterization of the molecular mechanisms involved in the quantitative life-history trait modifications of *Arabidopsis* triggered by CMV (currently underway in our laboratory) will further shed light on the role of these responses in tolerance.

The increased reproduction investment in infected individuals of accession group 1 conforms to predictions for highly virulent parasites [16]–[18]. For various animal-parasite systems increase in reproductive effort has been reported, estimated as parental care [59],[60], mating effort [24] or progeny production [23],[61]. Increased reproductive effort has also been reported in animals under strong predation pressure [62]–[64]. The few published plant reports also suggest that an increase in reproduction effort is a general response of plants to environmental stress. For instance, *Silene latifolia* plants infected by the castrating anther fungus *Microbotryum violaceum* developed a higher number of flowers than healthy ones. Nevertheless, this was interpreted as a response induced by the fungus in order to increase its transmission success rather than as a host tolerance mechanism [29],[30]. Higher resource allocation to reproduction also has been reported in plants under herbivory or abiotic stress [65]–[68]. Accelerated reproduction is also in agreement with theoretical predictions of host responses to minimize fitness losses caused by highly virulent parasites [20],[21]. The reduction of *RP* in infected plants of accession group 1 is in concordance with experimental evidence from animal host-parasite systems on faster reproduction when parasitised [23],[24]. Faster reproduction reduces exposure time to parasite infection and optimises host fitness, since reproduction success will be higher when the time of exposure to the parasite is shorter [16],[17]. In agreement with this hypothesis, earlier reproduction has been reported for predator-prey systems [62],[69],[70] or in plants under abiotic stress [37],[45],[65],[67]. Nevertheless, the small reduction of *RP* in infected plants of group 1 suggests that it will play a minor role in their tolerance to CMV infection as compared to modifications of resource allocation.

A major question in the analysis of life-history trait evolution is whether observed plastic responses are genetically determined [7]. Most experimental reports are inconclusive because do not include different host genotypes [6],[7]. We found genotype-specific life-history trait responses to CMV infection with significant genetic variation (heritability), and plant genotypes explained the largest fraction of the observed life-history traits variance. A genetic control of life-history responses has been reported also in plants under abiotic stress [36]. In addition, we observed significant differences in life-history traits between two experiments, where plants were inoculated at different developmental stages. These experiments did not differ for growth and reproduction efforts and life-history schedules in mock-inoculated plants (unpublished data). Therefore, the variation observed between experiments in CMV infected plants further indicates an important effect of the developmental stage at infection on the life-history responses to CMV infection.

In conclusion, our results are in agreement with modifications of life-history traits reported for parasitised animals, and with predictions from life-history theory. Thus, we provide empirical support for the general validity of theoretical predictions. This experimental approach shows that the capacity to modify life histories depends on the host genotype, and allows estimating quantitatively the genetic determinism of life-history trait plasticity. In addition, we were able to evaluate more precisely the role of life-history trait modification in defence against parasites by taking into account plant/virus genotype combinations where life-history traits were differentially modified.

## Materials and Methods

### Viral isolates, *Arabidopsis* accessions, and inoculations

CMV isolates Fny-CMV, belonging to subgroup I of CMV strains, and LS-CMV, belonging to subgroup II have been described and were derived from biologically active cDNA clones [71],[72]. De72-CMV, belonging to subgroup I, was initially derived from a field-infected plant of *Diplotaxis erucoides (Brassicaceae)* [73]. Isolates were multiplied in tobacco plants, virions from tobacco leaves were purified as described in Lot et al. [74] and viral RNA was extracted by virion disruption with phenol and sodium dodecyl sulphate.

Eighteen wild genotypes (accessions) of *Arabidopsis*, described in Pagan et al. [34] (see Table S1), were selected to include a broad amount of natural genetic variation of the species in Eurasia and in the Iberian Peninsula, which has been suggested as a Pleistocene glacial refuge for *Arabidopsis* [75]. Accessions were kindly obtained from Maarten Koornneef (Max Planck Institute for Plant breeding, Cologne, Germany) or were kept in the laboratory of Carlos Alonso-Blanco (CNB-CSIC, Madrid, Spain). The 18 accessions were initially multiplied simultaneously under the same greenhouse conditions to minimise maternal effects. For experiments, seeds were sown on filter paper soaked with water in plastic Petri dishes, and stratified in darkness at 4°C for 3 days before transferring for germination to a growth chamber (22°C, 14 h light and 70% relative humidity). Five day-old seedlings were planted in soil containing pots 10.5 cm of diameter, 0.43 l volume and grown in a greenhouse (25/20°C day/night, 16 h light).

The experimental design is described in detail in Pagán et al. [34]. Briefly, each accession was inoculated with the three CMV isolates. Ten individual plants per treatment, including mock-inoculated controls, were grown in a greenhouse in a completely randomised design. Three rosette leaves per plant were mechanically inoculated with 5 µl of a 100 µg/ml suspension of purified CMV RNA when rosettes presented 4–5 leaves (stages 1.04–1.05 in Boyes et al. [40]). In a second experiment, plants were inoculated when the inflorescence started bolting (first flower bud visible, growth stage 5.0/5.1 as in Boyes et al. [40]). Overall results were similar in both experiments and therefore, only the results of the first one are shown.

### Quantification of *Arabidopsis* life-history traits

Plants were harvested at complete senescence stage, and dry weight was determined after plants were maintained at 65°C until constant weight. The weights of rosettes (rosette weight, *RW*), inflorescence structures including seeds (inflorescence weight, *IW*) and seeds (seed weight, *SW*) were measured separately, and the above ground biomass (*BM*) was estimated as *RW* plus *IW.* Following Thompson and Stewart [43], rosette weight was used as an estimate of growth effort, inflorescence weight was taken as an estimate of total reproductive effort (reproductive structures plus seed output) and seed weight was used as an estimator of progeny production.

Two temporal parameters of *Arabidopsis* life cycle were quantified. Growth period span (*GP*) was measured as the time (days) elapsed between planting of seedlings on soil and opening of the first flower (stage 6.0 of Boyes et al. [40]). Reproductive period span (*RP*) was measured as the time (days) from the opening of the first flower to shattering of the first silique, which is the period dedicated to flower production (stage 8.0 of Boyes et al. [40]).

To quantify the effect of CMV infection on life-history traits, the value of each infected plant was divided by the mean value of the mock-inoculated plants of the same genotype.

### Statistical analyses

*RW*, *IW*, *SW* and *BM* and their various transformations, were homocedastic and were analysed using ANOVA. Data on *GP* and *RP* showed heterogeneity of variances and therefore differences in *GP* and *RP* among CMV isolates or *Arabidopsis* accessions were also tested by Kruskal-Wallis test. Since ANOVA comparisons of these data led to similar results and conclusions than Kruskal-Wallis test, for simplicity, only ANOVA analyses are shown.

All traits were compared among CMV isolates or *Arabidopsis* accessions within each experiment by two-way ANOVA using accession and isolate as factors in a complete model. To determine if there are differences in the traits among experiments, a complete three-way ANOVA model was used including accession, isolate and experiment as factors. To test if viral infection affected differentially host life-history traits, a complete three-way ANOVA model was used including accession, isolate, and life-history trait as factors. Differences between allometric groups were analysed by two way ANOVA using isolates and groups as factors. Significance of differences among classes within each factor was determined by Least Significant Difference (LSD) analyses. Accession, isolate, experiment, life-history trait and allometric group were considered as random effect factors in all ANOVAs. For each trait, the percentage of total variance explained by each factor was calculated by variance component (*VC*) analyses in the corresponding models described above. All of these comparisons were done for the raw untransformed data, and for ratios and differences between values of infected and mock-inoculated plants. The three analyses lead to the same conclusions. As allometric relationships are usually expressed as ratios, we present only the results of analyses using this transformation.

Broad-sense heritability (*h^2^_b_*) of the traits was estimated as the percentage of the total variance accounted by genetic (among accession) variance (*h^2^_b_*_ = _σ^2^_G_ /σ^2^_P_, where σ^2^_G_ is the genetic variance and σ^2^_P_ is the total phenotypic variance). On all plant traits, σ^2^_P_ and σ^2^_G_ were derived as variance components from univariate analyses for each viral isolate.

Correlations between variables were tested using Pearson coefficients. Analysis involving non-parametric variables were also done using Kendall's robust test and Spearman's correlation test, showing similar results. Linear regression equations were compared using ANOVA to test equality of slopes and intercepts. All statistical analyses were done using the statistical software package SPSS 13.0 (SPSS Inc., Chicago, USA).

## Supporting Information

Figure S1 Effect of CMV infection on rosette and inflorescence weight of *Arabidopsis* accessions. (A) Viral effect on rosette weight of plants estimated as *RW_i_/RW_m_*, where *i* and *m* denote infected and mock-inoculated plants, respectively. (B) Viral effect on inflorescence weight estimated as described for (A). Data are mean±standard errors of 10 replicates. The effect of infection is shown for LS-CMV (green), Fny-CMV (blue), and De72-CMV (red). Accessions are divided into allometry groups 1 and 2.
(1.82 KB TIF)

Table S1 Origin of *Arabidopsis thaliana* accessions analysed in this work.
(35 KB DOC)

Table S2 Statistical parameters of analysed host life-history traits.
(90 KB DOC)

Table S3 Two-way ANOVAs of *Arabidopsis* life-history traits responses to CMV infection, using accession and virus isolate as factors.
(44 KB DOC)

Table S4 Two-way ANOVAs of *Arabidopsis* life-history traits responses to CMV infection, using virus isolate and allometry group as factors.
(44 KB DOC)
