# Supplementary figures and images for: Host Responses in Life-History Traits and Tolerance to Virus Infection in Arabidopsis thaliana
Source: PLoS Pathog. 2008 Aug 15;4(8):e1000124. doi: 10.1371/journal.ppat.1000124 (PMC2494869; doi:10.1371/journal.ppat.1000124)

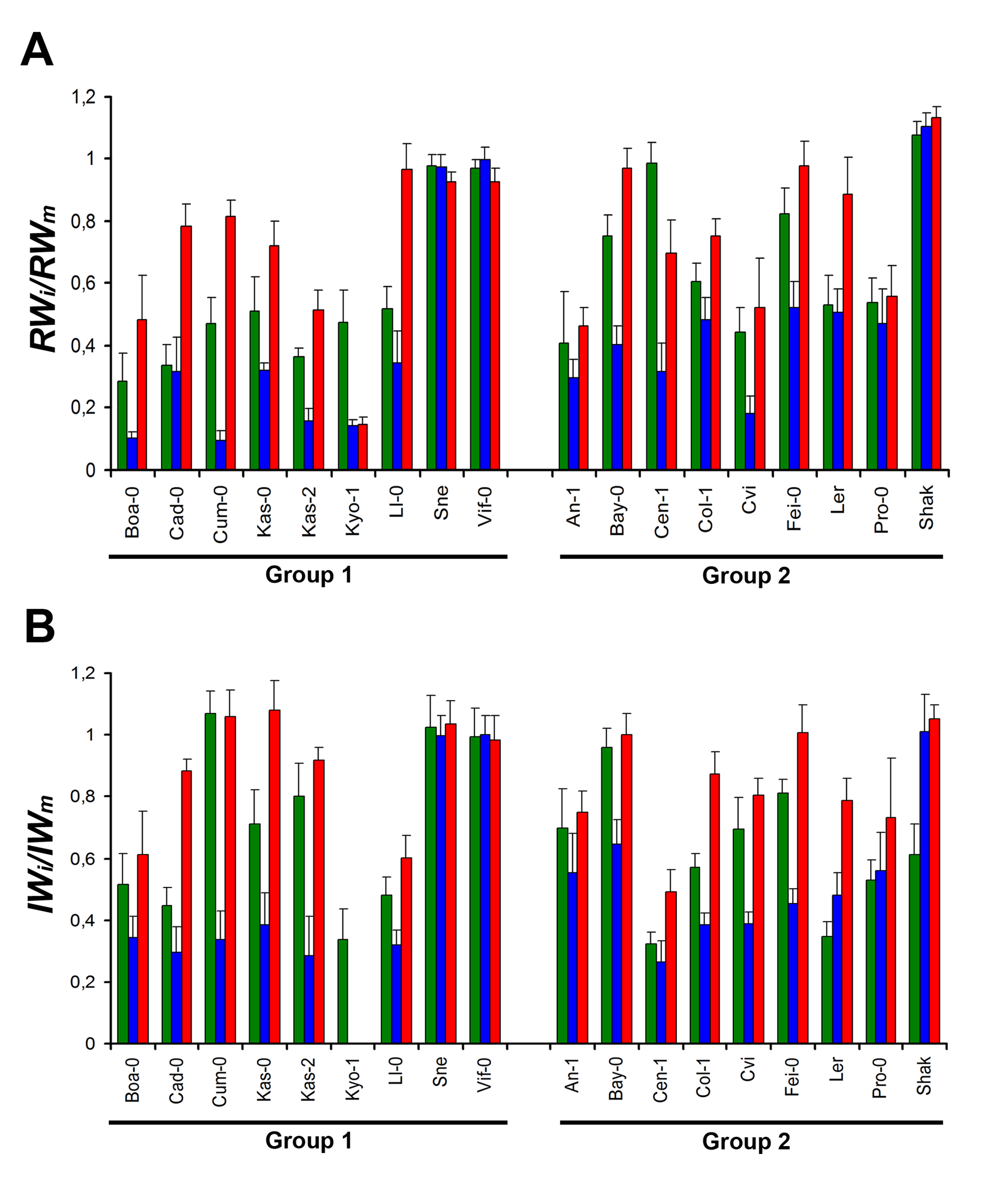

Supplement: Figure S1 — Effect of CMV infection on rosette and inflorescence weight of Arabidopsis accessions. (A) Viral effect on rosette weight of plants estimated as RWi/RWm, where i and m denote infected and mock-inoculated plants, respectively. (B) Viral effect on inflorescence weight estimated as described for (A). Data are mean±standard errors of 10 replicates. The effect of infection is shown for LS-CMV (green), Fny-CMV (blue), and De72-CMV (red). Accessions are divided into allometry groups 1 and 2. (1.82 KB TIF) [file ppat.1000124.s001.tif]
