# Supplementary material for: Host Responses in Life-History Traits and Tolerance to Virus Infection in Arabidopsis thaliana
Source: PLoS Pathog. 2008 Aug 15;4(8):e1000124. doi: 10.1371/journal.ppat.1000124 (PMC2494869; doi:10.1371/journal.ppat.1000124)
Supplement: Table S1 — Origin of Arabidopsis thaliana accessions analysed in this work. (35 KB DOC) [file ppat.1000124.s002.doc]

**Table S1.** Origin of *Arabidopsis thaliana* accessions analysed in this work.

| **Name** | **Origin** |
| --- | --- |
| An-1 | Amberes (Belgium) |
| Bay-0 | Bayreuth (Germany) |
| Boa-0 | Boadilla del Monte (Spain) |
| Cad-0 | Candelario (Spain) |
| Cen-1 | Centenera (Spain) |
| Col-1 | Columbia (Unknown) |
| Cum-0 | Cumbres Mayores (Spain) |
| Cvi | Cape Verde Islands |
| Fei-0 | Santa María da Feira (Portugal) |
| Kas-0 | Kashmir (India) |
| Kas-2 | Kashmir (India) |
| Kyo-1 | Kyoto (Japan) |
| L*er* | Landsberg (Poland) |
| Ll-0 | Llagostera (Spain) |
| Pro-0 | Proaza (Spain) |
| Shak | Shakdara (Tadjikistan) |
| Sne | Sierra Nevada (Spain) |
| Vif-0 | Villafáfila (Spain) |
