# Supplementary material for: Host Responses in Life-History Traits and Tolerance to Virus Infection in Arabidopsis thaliana
Source: PLoS Pathog. 2008 Aug 15;4(8):e1000124. doi: 10.1371/journal.ppat.1000124 (PMC2494869; doi:10.1371/journal.ppat.1000124)
Supplement: Table S2 — Statistical parameters of analysed host life-history traits. (90 KB DOC) [file ppat.1000124.s003.doc]

**Table S2.** Statistical parameters of analysed host life-history traits.

|  | **Isolate^5^** | ***GP*^6^** | ***GP_i_/GP_m_*^6^** | ***RP*^6^** | *RP_i_/RP_m_*^6^ | ***RW*^6^** | *RW_i_/RW_m_*^6^ | *IW*^6^ | *IW_i_/IW_m_*^6^ | | ***(IW_i_/RW_i_)/(IW_m_/RW_m_)*** **^6^** | |
| --- | --- | --- | --- | --- | --- | --- | --- | --- | --- | --- | --- | --- |
| **Acc. Mean^1^** | ***Mock*** | 40,26 ± 0.62 | - | 19.50 ± 0.41 | - | 0,44 ± 0.02 | - | 0,94 ± 0.03 | - | | - | |
|  | ***Fny*** | 44.12 ± 0.48 | 1.08 ± 0.01 | 13.98 ± 0.26 | 0.96 ± 0.04 | 0.13 ± 0.01 | 0.36 ± 0.02 | 0.39 ± 0.02 | 0.42 ± 0.02 | | 1.76 ± 0.09 | |
|  | ***De72*** | 42.33 ± 0.47 | 1.06 ± 0.01 | 14.13 ± 0.26 | 0.98 ± 0.04 | 0.38 ± 0.01 | 0.73 ± 0.02 | 0.75 ± 0.02 | 0.82 ± 0.02 | | 1.09 ± 0.05 | |
|  | ***LS*** | 42.86 ± 0.45 | 1.04 ± 0.01 | 14.18 ± 0.25 | 0.96 ± 0.04 | 0.21 ± 0.01 | 0.51 ± 0.02 | 0.59 ± 0.02 | 0.59 ± 0.02 | | 1.37 ± 0.09 | |
| **Acc. Min-Max^2^** | ***Mock*** | 13.0 - 78.5 | - | 12.41 - 27.40 | - | 0.03 - 1.13 | - | 0.44 - 1.48 | - | | - | |
|  | ***Fny*** | 13.9 - 80.7 | 0.94 - 1.26 | 12.33 - 25.11 | 0.43 - 1.90 | 0.01 - 0.25 | 0.09 - 1.11 | 0.17 - 0.87 | 0.26 - 1.01 | | 0.32 - 5.54 | |
|  | ***De72*** | 14.8 - 79.3 | 0.93 - 1.30 | 11.90 - 27.00 | 0.45 - 1.92 | 0.03 - 0.95 | 0.15 - 1.13 | 0.33 - 1.31 | 0.49 - 1.08 | | 0.12 - 1.97 | |
|  | ***LS*** | 13.9 - 79.5 | 0.89 - 1.18 | 12.10 -27.33 | 0.62 - 1.46 | 0.02 - 0.53 | 0.19 - 1.08 | 0.07 - 1.16 | 0.16 - 0.96 | | 0.27 - 2.98 | |
| ***CV*^3^** | ***Mock*** | 52 | - | 7 | - | 84 | - | 33 | - | | - | |
|  | ***Fny*** | 58 | 7 | 4 | 34 | 46 | 65 | 55 | 48 | | 60 | |
|  | ***De72*** | 55 | 7 | 9 | 40 | 80 | 32 | 50 | 34 | | 42 | |
|  | ***LS*** | 57 | 7 | 9 | 21 | 54 | 34 | 43 | 41 | | 52 | |
| **Acc. LSD^4^** | ***Mock*** | 5.89 | - | 2.42 | - | 0.18 | - | 0.37 | - | | - | |
|  | ***Fny*** | 4.63 | 0.16 | 1.44 | 0.42 | 0.10 | 0.18 | 0.20 | 0.22 | | 0.81 | |
|  | ***De72*** | 5.00 | 0.15 | 1.64 | 0.48 | 0.12 | 0.24 | 0.22 | 0.24 | | 0.50 | |
|  | ***LS*** | 4.00 | 0.12 | 1.55 | 0.43 | 0.12 | 0.43 | 0.21 | 0.23 | | 0.84 | |
| ***h^2^_b_*** | ***Mock*** | 0.96 | - | 0.31 | - | 0.88 | - | 0.53 | - | | - | |
|  | ***Fny*** | 0.96 | 0.14 | 0.12 | 0.31 | 0.21 | 0.56 | 0.47 | 0.39 | | 0.21 | |
|  | ***De72*** | 0.94 | 0.14 | 0.34 | 0.34 | 0.85 | 0.43 | 0.70 | 0.52 | | 0.40 | |
|  | ***LS*** | 0.97 | 0.23 | 0.36 | 0.15 | 0.53 | 0.11 | 0.64 | 0.46 | | 0.36 | |
|  |  |  |  |  |  |  |  |  |  | |  | |
|  | **Isolate^5^** | ***SW*^6^** | ***SW_i_/SW_m_*^6^** | ***IW-SW*^6^** | *(IW-SW)_i_/(IW-SW)_m_*^6^ | | ***[SW/(IW-SW)]_i_/[SW/(IW-SW)]_m_* ^6^** | | |  | | |
| **Acc. Mean^1^** | ***Mock*** | 0.13 ± 0.03 | - | 0.77 ± 0.04 | - | | - | | |  | |  |
|  | ***Fny*** | 0.06 ± 0.00 | 0.51 ± 0.04 | 0.33 ± 0.02 | 0.46 ± 0.08 | | 1.15 ± 0.08 | | |  | |  |
|  | ***De72*** | 0.10 ± 0.00 | 0.82 ± 0.05 | 0.67 ± 0.03 | 0.88 ± 0.08 | | 1.06 ± 0.07 | | |  | |  |
|  | ***LS*** | 0.08 ± 0.00 | 0.64 ± 0.05 | 0.50 ± 0.03 | 0.63 ± 0.08 | | 1.10 ± 0.06 | | |  | |  |
| **Acc. Min-Max^2^** | ***Mock*** | 0.01 - 0.38 | - | 0.36 - 1.35 | - | | - | | |  | |  |
|  | ***Fny*** | 0.00 - 0.20 | 0.13 - 1.20 | 0.16 - 0.67 | 0.10 - 2.19 | | 0.28 - 4.37 | | |  | |  |
|  | ***De72*** | 0.00 - 0.35 | 0.01 - 1.25 | 0.01 - 1.24 | 0.01 - 3.07 | | 0.29 - 1.81 | | |  | |  |
|  | ***LS*** | 0.00 - 0.37 | 0.04 - 1.37 | 0.07 - 1.03 | 0.16 - 1.17 | | 0.28 - 3.38 | | |  | |  |
| ***CV*^3^** | ***Mock*** | 83 | - | 37 | - | | - | | |  | |  |
|  | ***Fny*** | 110 | 59 | 41 | 71 | | 84 | | |  | |  |
|  | ***De72*** | 100 | 32 | 46 | 54 | | 27 | | |  | |  |
|  | ***LS*** | 119 | 51 | 53 | 37 | | 56 | | |  | |  |
| **Acc. LSD^4^** | ***Mock*** | 0.08 | - | 0.16 | - | | - | | |  | |  |
|  | ***Fny*** | 0.04 | 0.39 | 0.18 | 0.66 | | 0.68 | | |  | |  |
|  | ***De72*** | 0.05 | 0.58 | 0.19 | 0.74 | | 0.73 | | |  | |  |
|  | ***LS*** | 0.04 | 0.50 | 0.19 | 0.24 | | 0.77 | | |  | |  |
| ***h^2^_b_*** | ***Mock*** | 0.73 | - | 0.55 | - | | - | | |  | |  |
|  | ***Fny*** | 0.71 | 0.31 | 0.30 | 0.34 | | 0.39 | | |  | |  |
|  | ***De72*** | 0.75 | 0.19 | 0.68 | 0.37 | | 0.11 | | |  | |  |
|  | ***LS*** | 0.84 | 0.25 | 0.61 | 0.44 | | 0.21 | | |  | |  |

^1^ Mean value of each trait across accessions. Data are trait mean ± standard error.

^2^ Minimum and maximum mean values across accessions.

^3^ Coefficient of genetic variation, estimated as *CV_G_* = 100 x σ_G_ /$\bar{X}$, where $\bar{X}$ is the trait mean of accessions.

^4^ Least Significant Difference values.

**^5^ *Mock***: Mock-inoculated plants; ***Fny:*** Plants infected by Fny-CMV; ***De72:*** Plants infected by De72-CMV; ***LS:*** Plants infected by LS-CMV.

**^6^** ***GP***: Growth Period; ***GP_i_/GP_m_***: Viral effect on Growth Period; ***RP***: Reproductive Period; ***RP_i_/RP_m_****:*Viral effect on Reproductive Period; ***RW***: Growth Effort; ***RW_i_/RW_m_***: Viral effect on Growth Effort; ***IW***: Reproductive Effort; ***IW_i_/IW_m_***: Viral effect on Reproductive Effort. ***(IW_i_/RW_i_)/(IW_m_/RW_m_):*** Viral effect on Reproductive to Growth Effort relationship; ***SW***: Seed weight; ***SW_i_/SW_m_***: Viral effect on Seed Weight; ***IW-SW***: Reproductive structures weight; ***(IW-SW)_i_/(IW-SW)_m_****:* Viral effect on Reproductive structures weight; ***[SW/(IW-SW)]_i_/[SW/(IW-SW)]_m_***: Viral effect on Seed Weight to Reproductive structures weight relationship.
