# Supplementary material for: Host Responses in Life-History Traits and Tolerance to Virus Infection in Arabidopsis thaliana
Source: PLoS Pathog. 2008 Aug 15;4(8):e1000124. doi: 10.1371/journal.ppat.1000124 (PMC2494869; doi:10.1371/journal.ppat.1000124)
Supplement: Table S3 — Two-way ANOVAs of Arabidopsis life-history traits responses to CMV infection, using accession and virus isolate as factors. (44 KB DOC) [file ppat.1000124.s004.doc]

**Table S3.** Two-way ANOVAs of *Arabidopsis* life-history traits responses to CMV infection, using accession and virus isolate as factors.

|  |  |  | **Isolate** | | | |  | **Accession** | | | |  | **I x A** | | | |
| --- | --- | --- | --- | --- | --- | --- | --- | --- | --- | --- | --- | --- | --- | --- | --- | --- |
| Trait | *n* |  | *df* | F | *P* | % var |  | *df* | F | *P* | % var |  | *df* | F | *P* | % var |
| ***RW*** | 540 |  | 2 | 86.79 | 1x10^-5^ | 16.92 |  | 17 | 20.42 | 1x10^-5^ | 17.95 |  | 34 | 3.19 | 1x10^-5^ | 6.15 |
| ***IW*** | 540 |  | 2 | 87.59 | 1x10^-5^ | 22.22 |  | 17 | 17.49 | 1x10^-5^ | 19.14 |  | 34 | 4.80 | 1x10^-5^ | 16.67 |
| ***IW-SW*** | 540 |  | 2 | 86.98 | 1x10^-5^ | 2.93 |  | 20 | 8.73 | 1x10^-5^ | 5.39 |  | 40 | 3.49 | 1x10^-5^ | 3.29 |
| ***SW*** | 540 |  | 2 | 10.46 | 1x10^-5^ | 2.28 |  | 20 | 6.58 | 1x10^-5^ | 28.92 |  | 40 | 4.34 | 1x10^-5^ | 3.01 |
| ***IW/RW*** | 540 |  | 2 | 7.79 | 5x10^-4^ | 3.63 |  | 17 | 9.15 | 1x10^-5^ | 20 |  | 34 | 1.63 | 0.024 | 4.57 |
| ***SW/(IW-SW)*** | 540 |  | 2 | 4.70 | 0.009 | 4.72 |  | 20 | 11.04 | 1x10^-5^ | 48.44 |  | 40 | 2.24 | 0,005 | 6.65 |
| ***GP*** | 540 |  | 2 | 0.31 | 0.737 | - |  | 17 | 5.00 | 1x10^-5^ | 18.1 |  | 34 | 1.76 | 0.011 | 8.35 |
| ***RP*** | 540 |  | 2 | 0.09 | 0.918 | - |  | 17 | 9.49 | 1x10^-5^ | 17.27 |  | 34 | 2.16 | 8x10^-4^ | 9.39 |
| ***GP+RP*** | 540 |  | 2 | 2.04 | 0.131 | - |  | 17 | 4.27 | 1x10^-5^ | 12.12 |  | 34 | 1.12 | 0.005 | 6.06 |

Traits (ratios between CMV-infected and mock-inoculated plants) are listed on the left. ***n*:** number of observations. *df*: degrees of freedom. ***F*:** *F*-value from the type III sum of squares ANOVA for each factor and *P* is the estimated probability of obtaining this *F*-value under the null hypothesis. ***% var*:** percentage of trait variance explained by each factor.
