# Supplementary material for: Host Responses in Life-History Traits and Tolerance to Virus Infection in Arabidopsis thaliana
Source: PLoS Pathog. 2008 Aug 15;4(8):e1000124. doi: 10.1371/journal.ppat.1000124 (PMC2494869; doi:10.1371/journal.ppat.1000124)
Supplement: Table S4 — Two-way ANOVAs of Arabidopsis life-history traits responses to CMV infection, using virus isolate and allometry group as factors. (44 KB DOC) [file ppat.1000124.s005.doc]

**Table S4.** Two-way ANOVAs of *Arabidopsis* life-history traits responses to CMV infection, using virus isolate and allometry group as factors.

|  |  |  | **Isolate** | | | |  | **Group** | | | |  | **I x G** | | | |
| --- | --- | --- | --- | --- | --- | --- | --- | --- | --- | --- | --- | --- | --- | --- | --- | --- |
| Trait | *n* |  | *df* | F | *P* | % var |  | *df* | F | *P* | % var |  | *df* | F | *P* | % var |
| ***RW*** | 540 |  | 2 | 5.57 | 0.009 | 16.92 |  | 1 | 5.07 | 0.001 | 11.11 |  | 2 | 4.00 | 0.017 | 0.97 |
| ***IW*** | 540 |  | 2 | 16.53 | 1x10^-5^ | 23.03 |  | 1 | 4.28 | 0.009 | 19.45 |  | 2 | 15.58 | 1x10^-5^ | 4.32 |
| ***IW-SW*** | 540 |  | 2 | 60.84 | 1x10^-5^ | 3.39 |  | 1 | 1.94 | 0.231 | - |  | 2 | 4.23 | 0.015 | 2.12 |
| ***SW*** | 540 |  | 2 | 7.76 | 5x10^-4^ | 2.28 |  | 1 | 13.12 | 3x10^-4^ | 5.51 |  | 2 | 3.44 | 0.026 | 1.91 |
| ***IW/RW*** | 540 |  | 2 | 8.21 | 3x10^-4^ | 1.85 |  | 1 | 13.54 | 3x10^-4^ | 5.26 |  | 2 | 5.25 | 0.007 | 1.65 |
| ***SW/(IW-SW)*** | 540 |  | 2 | 1.37 | 0.256 | - |  | 1 | 11.87 | 6x10^-4^ | 61.76 |  | 2 | 1.08 | 0.341 | - |
| ***GP*** | 540 |  | 2 | 2.07 | 0.287 | - |  | 1 | 1.25 | 0.294 | - |  | 2 | 4.90 | 0.008 | 5.88 |
| ***RP*** | 540 |  | 2 | 2.00 | 0.137 | - |  | 1 | 7.94 | 1x10^-3^ | 5.11 |  | 2 | 3.94 | 0.020 | 1.23 |
| ***GP+RP*** | 540 |  | 2 | 1.97 | 0.331 | - |  | 1 | 7.45 | 2x10^-3^ | 3.21 |  | 2 | 4.89 | 0.007 | 1.45 |

Traits (ratios between CMV-infected and mock-inoculated plants) are listed on the left. ***n*:** number of observations. *df*: degrees of freedom. ***F*:** *F*-value from the type III sum of squares ANOVA for each factor and *P* is the estimated probability of obtaining this *F*-value under the null hypothesis. ***% var*:** percentage of trait variance explained by each factor.
